# Whole-Genome Sequencing Identifies Potentially Causative Variants in *SOX30*, *AKAP4*, and *RNF220* in Non-Obstructive Azoospermia

**DOI:** 10.3390/ijms27167230

**Published:** 2026-08-13

**Authors:** Razieh Ebrahimi Askari, Agnieszka Malcher, Fateme Sefid, Sara Sadeghzadeh, Rim Ibrahim, Krzysztof Pastuszak, Aashish Srivastava, Seyed Mehdi Kalantar, Serajoddin Vahidi, Nasrin Ghasemi, Maciej Kurpisz

**Affiliations:** 1 Department of Medical Genetics, Faculty of Medicine, Shahid Sadoughi University of Medical Sciences, Yazd 8916978477, Iran; mgnebrahimi@gmail.com (R.E.A.); kalantarsm@ystp.ac.ir (S.M.K.); ghasemi@ssu.ac.ir (N.G.); 2 Institute of Human Genetics, Polish Academy of Sciences, 60-479 Poznan, Poland; agnieszka.malcher@igcz.poznan.pl (A.M.); rim.ibrahim@igcz.poznan.pl (R.I.); 3 Department of Molecular Medicine, Faculty of Medicine, Shahid Sadoughi University of Medical Sciences, Yazd 8916978477, Iran; sefid.fateme@yahoo.com (F.S.); sadeghzadehsara3@gmail.com (S.S.); 4 Department of Algorithms and System Modelling, Gdansk University of Technology, 80-233 Gdansk, Poland; krzysztof.pastuszak@pg.edu.pl; 5 Department of Translational Oncology, Medical University of Gdansk, 80-210 Gdansk, Poland; 6 Centre of Biostatistics and Bioinformatics, Medical University of Gdansk, 80-210 Gdansk, Poland; 7 Genome Core Facility, Department of Clinical Science, Haukeland University Hospital, 5021 Bergen, Norway; aashish.srivastava@uib.no; 8 Abortion Research Center, Yazd Reproductive Sciences Institute, Shahid Sadoughi University of Medical Sciences, Yazd 8916877391, Iran; 9 Andrology Research Center, Yazd Reproductive Sciences Institute, Shahid Sadoughi University of Medical Sciences, Yazd 8916877391, Iran; vahidi.seraj@gmail.com

**Keywords:** infertility, non-obstructive azoospermia, *SOX30*, *AKAP4*, *RNF220*, whole-genome sequencing

## Abstract

More than 70% of non-obstructive azoospermia (NOA) cases remain idiopathic, and the underlying genetic causes need to be investigated. In this study, we aimed to identify genes and variants associated with the etiology of NOA. Two NOA patients from consanguineous families, with normal karyotypes and no Y-chromosome microdeletions, were selected for whole-genome sequencing (WGS). Candidate variants were validated by Sanger sequencing. Structural protein modeling and assessment of mutation-induced changes in molecular interactions were performed using SWISS-MODEL and AlphaFold. We identified a candidate homozygous missense variant (c.899C>T, p.(Ser300Phe); rs375931805) in *SOX30* in one patient; this transcription factor is highly expressed in the testis. In the second patient, a homozygous missense variant (c.1472C>T, p.(Thr491Met); rs746902232) in *RNF220* and a hemizygous missense variant (c.2354A>G, p.(Gln785Arg) in the X-linked *AKAP4* gene were simultaneously detected. Previous studies have shown that *SOX30* knockout in mice leads to meiosis I arrest and impaired spermiogenesis. *AKAP4* is exclusively expressed in the testis, and missense variants have been reported in different forms of male infertility. The co-occurrence of variants in *AKAP4* and *RNF220* may suggest an oligogenic etiology of NOA and contribute to the phenotypic variability associated with *AKAP4* variants.

## 1. Introduction

Male infertility accounts for approximately 50% of all infertility cases. Among its various causes is azoospermia, characterized by the complete absence of spermatozoa in the ejaculate. Azoospermia affects about 1% of men and 10–15% of infertile men [1].

Non-obstructive azoospermia (NOA) is the most severe form of male infertility. It is caused by intrinsic testicular failure and impaired spermatogenesis. Most NOA cases have primary testicular failure, resulting from defects that impair the initiation or progression of spermatogenesis. Some men develop secondary testicular failure due to hypothalamic-pituitary axis deficiency or other pre-testicular causes, such as developmental abnormalities [2]. NOA is frequently associated with underlying genetic defects such as chromosomal abnormalities, Y chromosome microdeletions, or rare monogenic variants. After excluding acquired causes, more than 70% of NOA cases remain idiopathic. Identifying its genetic causes is vital for the diagnosis, prognosis, and potential therapeutic interventions [3].

Whole-genome sequencing (WGS) and whole-exome sequencing (WES) are the leading technologies to detect genetic variants in idiopathic NOA cases. These approaches have been applied in many studies and have identified novel genes and variants related to the NOA phenotype [4,5]. By comparison, WGS provides a more comprehensive view of the genome and can detect a wider range of genetic variations, including non-coding genetic, structural, and copy number variations (CNVs), as well as repeat expansions. WGS is also a more effective method for sequencing complex regions of the genome and even better for capturing protein-coding regions [6]. WGS has emerged as a powerful tool for unraveling the genetic etiology of NOA, especially in consanguineous families [4,5].

Consanguinity, observed frequently in specific populations such as the Iranian, raises the probability of offspring inheriting homozygous variants. This genetic phenomenon can shed light on rare genes and variants responsible for recessive diseases [5].

In this study, we performed WGS on 2 patients with NOA, both from consanguineous families with first-cousin parents, to identify rare potentially causative variants involved in spermatogenesis and NOA. We identified candidate homozygous missense variants in *SOX30* and *RNF220*, as well as a hemizygous variant in *AKAP4*, that may contribute to NOA.

## 2. Results

**Figure 1:**
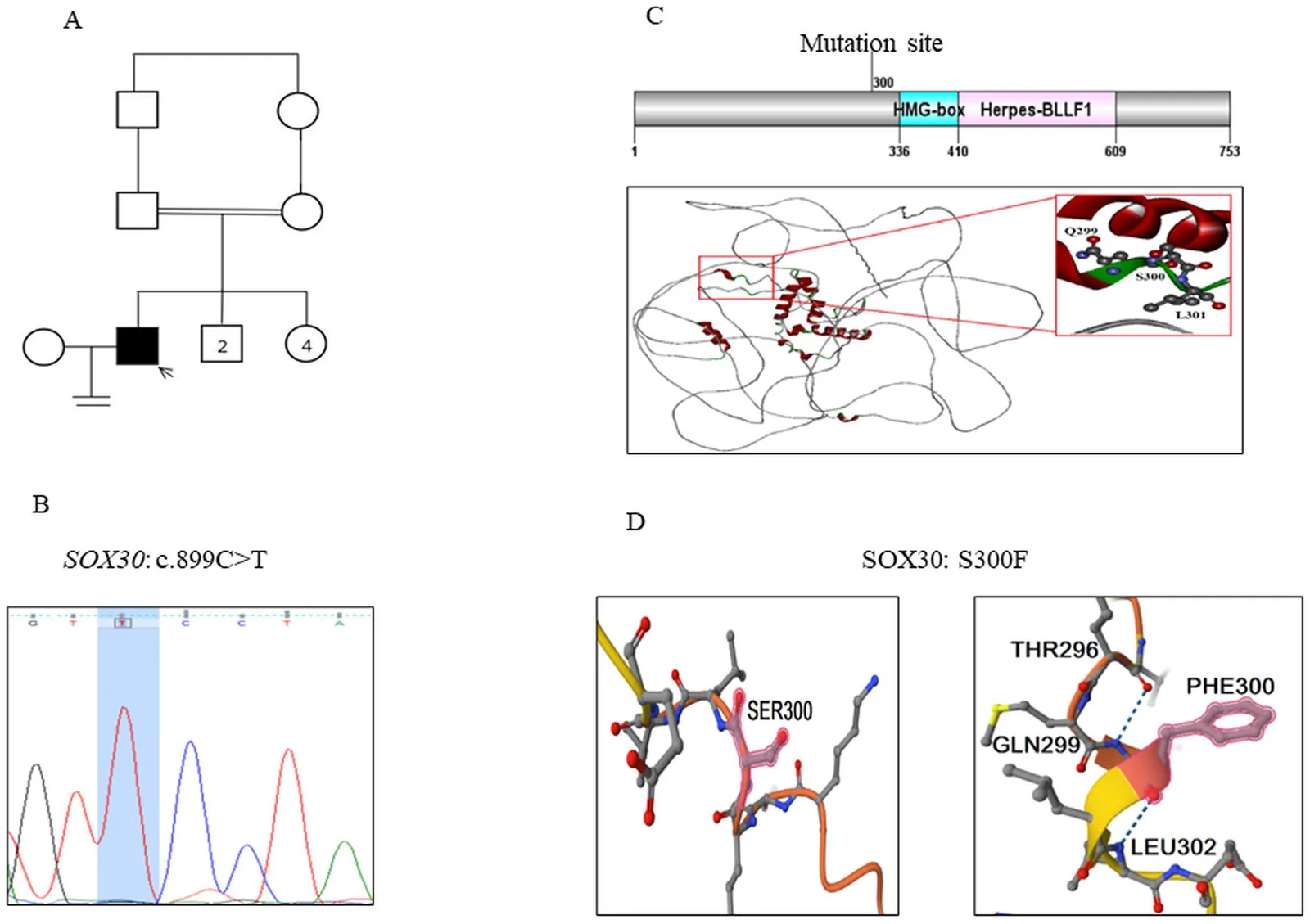
Pedigree of patient AZO-3, protein structural modeling, and mutation-induced changes in molecular interactions. (**A**) Pedigree of patient AZO-3; the arrow indicates the proband. (**B**) Sanger sequencing chromatogram validating the *SOX30* candidate variant c.899C>T. (**C**) Structural modeling of SOX30 protein. Diagram of SOX30 with the indicated domains. Numbers below represent amino acid positions in the primary structure. Above, the position of the mutation of interest is shown. The domain architecture includes the HMG-box (residues 336–410) and the Herpes-BLLF1-like region (residues 410–609). Below this scheme, the 3D structural model of SOX30 is shown. The inset shows the position of S300. (**D**) Comparative structural analysis of the wild-type and mutant SOX30 protein revealing potential mechanisms of mutation-induced perturbation. The SOX30-S300F mutation introduced significant structural rearrangements in the local loop region. While no hydrogen bonds were observed at this site in the wild-type structure, the mutant structure exhibits two novel hydrogen bonds: one between the backbone oxygen of Thr296 and the backbone nitrogen of Gln299, and another between the backbone oxygen of Gln299 and the backbone nitrogen of Leu302.

**Figure 2:**
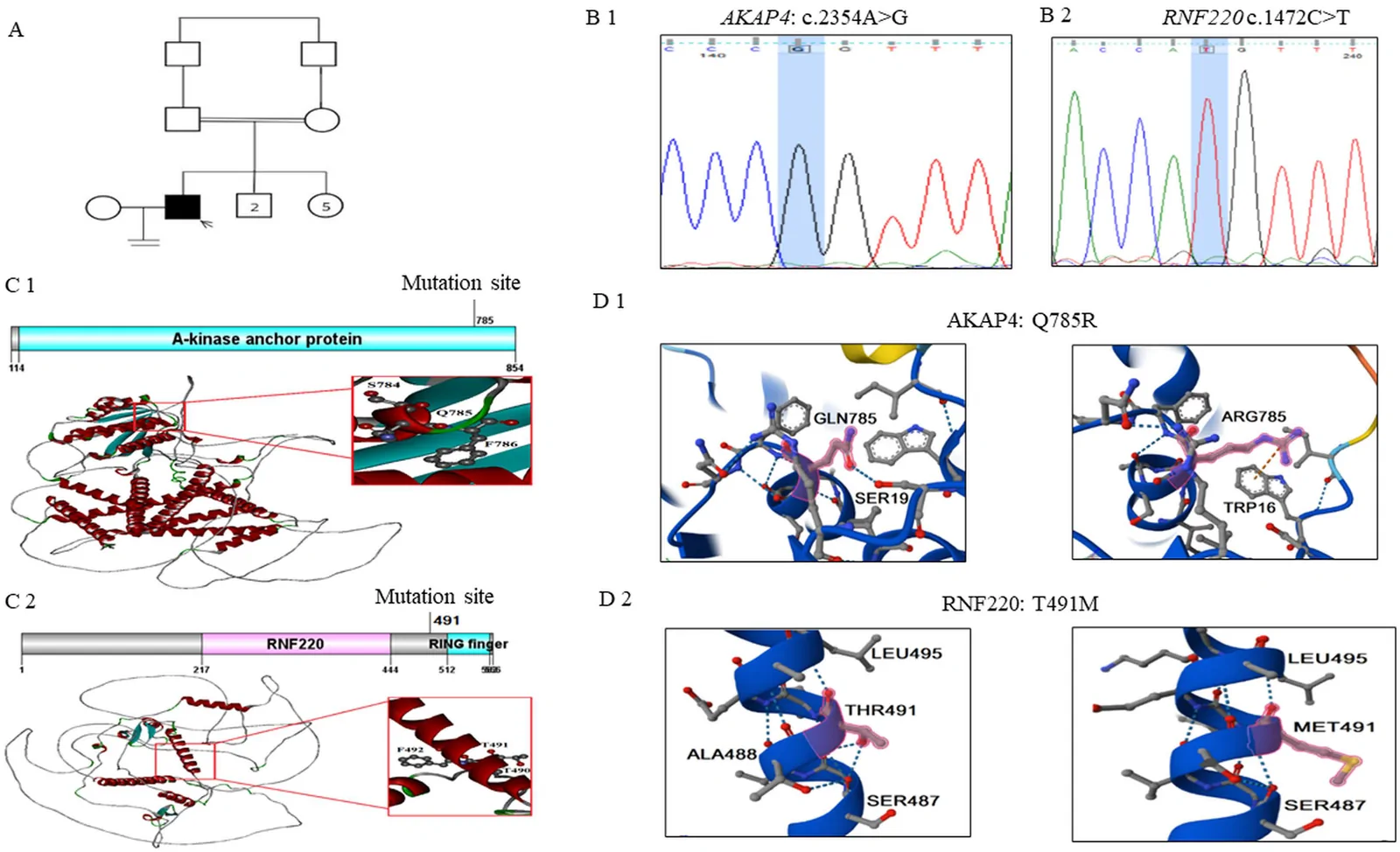
Pedigree of patient AZO-8, protein structural modeling, and mutation-induced changes in molecular interactions. (**A**) Pedigree of patient AZO-8; the arrow indicates the proband. (**B1**,**B2**) Sanger sequencing chromatograms validating the identified variants. (**B1**) Validation of the *AKAP4* c.2354A>G variant. (**B2**) Validation of the *RNF220* c.1472C>T variant. (**C1**,**C2**) Structural modeling of the AKAP4 and RNF220 proteins. Diagram of the proteins with the indicated domains. Numbers below represent amino acid positions in the primary structures. Above, positions of mutations of interest are shown. Below these schemes, the 3D structural models are shown. The insets show the position of the mutations: (**C1**) AKAP4: Interval 14-854 of the AKAP4 sequence belongs to the A-kinase anchor protein 110 kDa. (**C2**) RNF220: Interval 512-563 of the RNF220 sequence belongs to the RING finger. (**D1**,**D2**) Comparative structural analysis of the wild-type and mutant proteins revealing potential mechanisms of mutation-induced perturbation. (**D1**) AKAP4 Q785R: The wild-type interactions are characterized by a hydrogen bond between the OG atom of Ser19 and the OE1 atom of Gln785, which is absent in the mutant. This loss is compensated by the formation of a distinct cation-π interaction between the positively charged guanidinium group of Arg785 and the indole ring of Trp16. (**D2**) RNF220 T491M: In the wild-type, Thr491 engages in three hydrogen bonds—two via its side-chain OG1 atom (with Ala488/O and Ser487/O) and one via its backbone oxygen (with Leu495/N). In the Met491 mutant, the inability of the methionine side chain to form hydrogen bonds leads to the loss of the two side-chain interactions. The structure is stabilized by retention of the Met491/O...Leu495/N bond and the formation of a new hydrogen bond between the Met491 backbone nitrogen and the Ser487 backbone oxygen, indicating a local structural rearrangement.

In this study, we performed WGS on two patients from separate consanguineous families to identify potential genetic causes related to male infertility and impaired spermatogenesis (Figure 1A and Figure 2A).

### 2.1. Clinical Characteristics and Microsurgical Testicular Sperm Extraction (microTESE) Findings

Patient AZO-3 was a 33-year-old man who presented to the infertility clinic after 10 years of primary infertility. NOA was confirmed by two independent semen analyses and a comprehensive urological evaluation that excluded obstructive azoospermia. Physical examination was normal and testicular volume (>15mL) was within normal range. Hormonal evaluation revealed elevated FSH levels along with markedly reduced testosterone (Table 1). Histopathological subtyping was not available, as a testicular biopsy was not performed.

Patient AZO-8 was a 28-year-old man who was referred to the infertility clinic after 2 years of primary infertility. Physical examination and testicular volume (>15 mL) were both normal. The diagnosis of NOA was established based on repeated semen analyses, comprehensive urological evaluation, and further supported by microTESE findings. Hormonal assessments showed FSH and LH levels within normal ranges but reduced testosterone (Table 1). Histological evaluation of the microTESE sample from the right testis revealed the presence of primary spermatocytes, spermatids, and spermatozoa along with normal Sertoli and Leydig cell populations. These findings were consistent with hypospermatogenesis. Although spermatozoa were successfully retrieved, subsequent ICSI treatment of 24 oocytes resulted in only 5 embryos, and none of the transferred embryos led to a clinical pregnancy. Importantly, the female partner underwent thorough clinical and laboratory evaluations, all of which were within normal limits, indicating that her reproductive and fertilization potential was not compromised.

### 2.2. Identified Potential Causative Variants

In patient AZO-3, we identified an ultrarare homozygous missense variant (c.899C>T) in the *SOX30* (SRY-Box Transcription Factor 30) gene, leading to the substitution of Serine with Phenylalanine at position 300 (p.Ser300Phe) (Table 2). *SOX30* belongs to the SOX (SRY-related High Motility Group (HMG) box) gene family and is mainly expressed in the testis with a 138.9 transcripts per million (TPM) value (https://gtexportal.org/home/gene/SOX30, accessed on 6 February 2026). The GTEx expression data used in this study are derived from healthy human tissue samples and not from our patients. The VCF output for patient AZO-3 is provided as Table S1.

In patient AZO-8, we detected a hemizygous variant (c.2354A>G) in the X-linked gene *AKAP4* and a homozygous variant (c.1472C>T) in the *RNF220* gene (Table 2). The *AKAP4* gene encodes A kinase anchoring protein 4, which is exclusively expressed in the testis, with a 493 TPM value (https://gtexportal.org/home/gene/AKAP4, accessed on 6 February 2026). The *RNF220* (Ring Finger Protein 220) gene encodes an E3 ubiquitin-protein ligase expressed in many tissues and organs, including the testis, with a 21.63 TPM (https://gtexportal.org/home/gene/RNF220, accessed on 6 February 2026). The VCF output for patient AZO-8 is provided as Table S2.

### 2.3. Structural Protein Modeling and Characterization of Mutation-Induced Changes in Molecular Interactions

Structural modeling of the SOX30 protein indicated that the location of the c.899C>T (p.Ser300Phe) variant is near the N-terminal region of the HMG-box domain (Figure 1C). Comparative structural analysis revealed a notable alteration in the interaction network at the mutation site. The SOX30-S300F substitution introduced significant structural rearrangements within the local loop region (Figure 1D).

The AKAP4-Q785R is located within the A-kinase anchoring protein domain (2C1). Comparative analysis of the AKAP4 wild-type and Q785R mutant structures highlighted significant changes in the local bonding pattern (Figure 2(D1)).

Comparative analysis of the RNF220 wild-type and mutant structures revealed a shift from side-chain to backbone hydrogen bonding upon the T491M substitution (Figure 2(C2,D2)).

## 3. Discussion

In patient AZO-3, we found a homozygous missense variant (c.899C>T, p.Ser300Phe) in *SOX30*, a transcription factor belonging to the SRY-related HMG box (SOX) family. Structural modeling indicated that the SOX30-S300F substitution led to the gain of two novel hydrogen bonds (Thr296→Gln299 and Gln299→Leu302). This suggests that the mutation stabilizes an alternative backbone conformation, potentially rigidifying a flexible loop. Such rearrangement could alter the dynamics or interaction surfaces of the SOX30 DNA-binding domain, with functional consequences in its transcriptional activity. However, due to the unavailability of parental samples for segregation analysis and the lack of functional studies, these findings should be interpreted with caution and considered preliminary findings.

Previous studies have shown that *SOX30* expression in mice is restricted to spermatocytes and round spermatids, and its inactivation leads to spermiogenic arrest and male sterility. Deficient mice exhibit abnormal chromocenter formation, increased germ cell apoptosis, and disrupted acrosome and axoneme development [7]. *SOX30* is expressed in meiotic spermatocytes and post-meiotic haploids, initiating the transcription of genes during late meiosis and spermiogenesis [8]. However, *SOX30* expression was also observed in spermatogonia, Leydig cells, and Sertoli cells, and its deficiency caused spermatocyte arrest at the early phase of meiosis I, with nearly no normally developing secondary spermatocyte [9]. These findings support the role of *SOX30* in spermatogenesis and suggest that it may be a potential candidate gene for human NOA.

In humans, *SOX30* promoter hypermethylation is associated with non-obstructive azoospermia [10]. In a case–control study involving 15 infertile males and 15 healthy controls, a significant difference in the expression of *SOX30* has been reported, indicating a possible dysregulation in infertile males [11]. In another study, Miyamoto et al. sequenced the coding region of the *SOX30* gene in 138 Japanese men with SCOS. Among the six identified SNPs, only one was predicted to be disease-causing based on in silico analysis. However, there were no significant differences in the frequency or distribution of these SNPs compared to the control group (99 fertile men) [12].

The variant identified in patient AZO-3 (c.899C>T) in *SOX30* has been previously reported in ClinVar as a variant with uncertain significance and unknown affected status (Variation ID: 2271360). Another *SOX30* variant, c.1550C>G (p.Ser517Cys), has been reported as likely pathogenic for male infertility (Variation ID: 812722). The AZO-3 patient did not undergo microTESE, and there is no information concerning the histopathology.

A recent study reported six heterozygous *SOX30* variants in a cohort of 620 individuals with NOA and provided functional evidence that impaired SOX30 activity may affect spermatogenesis [13]. In our study, the identification of a homozygous missense variant in *SOX30* (p.Ser300Phe) in a consanguineous family adds further support to the potential involvement of *SOX30* alterations in NOA and expands the currently reported variant spectrum.

We identified a homozygous variant (c.1472C>T, p.Thr491Met) in *RNF220* and, interestingly, a hemizygous variant (c.2354A>G, p.Gln785Arg) in the X-linked gene *AKAP4* in patient AZO-8. The AKAP4-Q785R substitution induces a fundamental shift in the local molecular interactions within the loop containing residue 785, replacing a polar hydrogen bond (Gln785–Ser19) with a strong non-covalent cation–π interaction (Arg785–Trp16). This substitution not only alters the chemical nature of the binding but also potentially reorients the local backbone and side chains, which could have significant implications for the structural integrity and function of the AKAP4 domain.

*AKAP4* is exclusively expressed in germ cells [14]. It is crucial for binding cyclic-AMP dependent protein kinase A (PKA) to its substrates for protein phosphorylation and for organizing the sperm fibrous sheath (FS) skeletal structure [15]. Previous studies identified several *AKAP4* variants related to male infertility. The hemizygous missense variant c.454T>C (p.S152P) is associated with asthenozoospermia [16]. The hemizygous missense variant c.1285C >T (p.R429C) has been shown to cause dysplastic fibrous sheath, reduced sperm motility, and severe oligozoospermia, while a different missense change involving the same amino acid residue (c.1286G>A/ p.R429H) segregates with NOA in two siblings with meiotic arrest. An equivalent knock-in mouse model (Akap4R428H) characterized by flagella FS abnormality, reduced sperm count, and motility [16,17]. High sperm DNA fragmentation index (H-DFI) has been associated with reduced fertilization rates, impaired embryo development, lower implantation, and decreased pregnancy rates [18]. In high DFI sperm, decreased AKAP4/PKA signaling impairs protein phosphorylation events, blocks capacitation, and reduces the acrosome reaction, ultimately resulting in lower fertilization rates [19].

Different AKAP4 variants have been associated with variable reproductive phenotypes, ranging from asthenozoospermia and severe oligozoospermia to NOA with meiotic arrest. Similar to previously reported siblings carrying the AKAP4 p.R429H variant, our patient AZO-8 also presented with NOA; however, histological evaluation revealed hypospermatogenesis rather than comprehensive meiotic arrest.

The *RNF220*-T491M substitution alters the hydrogen-bonding pattern. The loss of the polar threonine side chain eliminates two key hydrogen bonds, which could potentially destabilize the local structure. However, the structure compensates for this by forming a new backbone hydrogen bond (Met491/N...Ser487/O) while maintaining the critical Met491/O...Leu495/N interaction. This suggests a structural adaptation by network transitions from side-chain-dominated (in wild-type) to backbone-dominated (in mutant). This alteration may affect the flexibility and conformational dynamics of this loop, which could influence the protein’s interaction with its substrates.

The *RNF220* gene encodes a RING finger E3 ubiquitin ligase involved in various cellular processes, including protein turnover, signal transduction, and DNA damage response. It promotes SIN3B ubiquitination and proteasomal degradation. Guo et al. identified a homozygous missense mutation (c.56C>T/ p.P19L) in the *RNF220* gene in an infertile patient with small-headed sperm. They argued that elevated levels of *SIN3B* caused excessive chromatin condensation and small-headed sperm [20].

The co-occurrence of *RNF220* and *AKAP4* variants in patient AZO-8 supports potential oligogenic contributions to male infertility. Previous studies have suggested that oligogenic inheritance may contribute to male infertility [21]. However, this observation alone does not establish an oligogenic mechanism, and further studies are required to determine the contribution of these specific variants. Although spermatozoa were successfully obtained from patient AZO-8, the subsequent ICSI procedure resulted in a low fertilization rate and ultimately no clinical pregnancy, despite the female partner’s normal clinical and laboratory evaluations. Therefore, the identified variants in the *AKAP4* and *RNF220* genes may not only contribute to failed natural conception but also to poor assisted reproduction outcomes.

Both patients showed reduced testosterone levels, which may reflect impaired testicular function; however, the relationship between these hormonal alterations and the identified variants remains unclear.

The major limitation of this study was the unavailability of other family members for segregation analysis and the lack of microTESE results for patient AZO-3.

Further studies, including functional analyses, family segregation, and cohort studies, are needed to validate the pathogenicity of these genes and variants and their contribution to NOA.

## 4. Materials and Methods

### 4.1. Patient Selection and Sample Preparation

Two men with primary infertility who were referred to the infertility clinic were selected from consanguineous families. Both patients had normal karyotype, no microdeletions of the Y chromosome, and no history of drug abuse, varicocele, testicular torsion, orchitis, and cryptorchidism were selected from consanguineous families. NOA was confirmed based on at least two semen analyses demonstrating azoospermia after centrifugation, in combination with comprehensive urological evaluation, including detailed medical history and physical examination.

Whole blood (5–10 mL) was collected in sterile, single-use EDTA-containing tubes for the subsequent genomic DNA isolation. DNA was extracted using the High Pure PCR Template Preparation Kit (Roche Diagnostics GmbH, Mannheim, Germany) according to the manufacturer’s protocol. The purity and concentration of extracted DNA were assessed using a NanoDrop 1000 spectrophotometer (Thermo Scientific, Waltham, MA, USA) and the Quantus fluorometer (Promega, Madison, WI, USA). Purified DNA samples were stored at −20 °C until use.

MicroTESE was performed following standard surgical procedures. The histological phenotype was classified based on microTESE findings as Sertoli cell-only syndrome (SCOS), maturation arrest, or hypospermatogenesis.

### 4.2. Whole-Genome Sequencing and Variant Prioritization

The genomic DNA samples were sequenced with the Illumina NovaSeq X platform (Illumina, Inc., San Diego, CA, USA), achieving an average depth of ×30 (~120 Gbp). Raw FASTQ files were processed using cgpmap, and quality control was performed using FastQC (v0.12.0) before processing. Reads were aligned to the human reference genome (GRCh38) using BWA-MEM. Duplicate reads were marked with biobambam2, and post-processing was managed by SAMtools (v1.21).

DeepVariant (v1.6.0) was used for variant calling, retaining only variants with PASS status from its internal filters. VCFtools (v0.1.16) was employed to filter variants based on a minimum quality score (QUAL) ≥ 30, mean depth of coverage (DP) ≥ 10×, and a genotype call rate of ≥90%. Functional annotation was performed with Ensembl Variant Effect Predictor (VEP, release 112), selecting representative transcripts based on predefined criteria. Supplementary pathogenicity scores from dbNSFP version 4.4a were integrated and annotated using SnpSift (v5.2).

Rare variants were prioritized by excluding those with a gnomAD genomes allele frequency (gnomADg_AF) ≥ 0.01. Variants without gnomAD annotations or missing frequency values were preserved to potentially identify novel rare alleles.

Subsequent analyses focused on variant types, inheritance patterns, and Mutation Taster predictions. Expression levels were evaluated using the Genotype-Tissue Expression project (GTEx v7). The functional relevance of the candidate variants related to fertility and spermatogenesis was explored through PubMed searches using the Gene ID combined with keywords such as “spermatogenesis,” “infertility,” “azoospermia,” and “reproductive biology.”

Additionally, CNVs were detected using ClinSV (v1.0) and assessed alongside single-nucleotide variants (SNVs) and indels for a comprehensive analysis of genomic alterations associated with male infertility.

### 4.3. Sanger Sequencing

To validate the candidate variants identified in two patients (AZO-3 and AZO-8) through whole-genome sequencing, Sanger sequencing was performed using an ABI 3500XL Genetic Analyzer (Applied Biosystems, Waltham, MA, USA). Analyses were performed with Finch TV software (v1.4.0). PCR amplification was carried out using the following primers (5′–3′): *SOX30* (forward: GATCCCTTTGACGCTCCAC; reverse: GCCCTGAAAACAATCAGTTCC; amplicon size: 313 bp), *AKAP4* (forward: ACAAGCAGCCTCGGCAAATA; reverse: AGGGTCACGGATGAAATGCG; amplicon size: 475 bp), and *RNF220* (forward: AAATGGTTCCCTGGTATCTGG; reverse: CAGGCTCAGGCATAAGCACA; amplicon size: 613 bp).

### 4.4. Structural Protein Modeling and Characterization of Mutation-Induced Changes in Molecular Interactions

The SOX30 protein sequence (accession No. NP_848511.1), the RNF220 protein sequence (accession No. NP_001363417.1), and the AKAP4 protein sequence (accession No. NP_003877.2) were obtained from the NCBI database (http://www.ncbi.nlm.nih.gov/protein, accessed on 7 January 2026) and saved in FASTA format for further analyses. Structural modeling was performed using the SWISS-MODEL server with the AlphaFold v2 method, accessible via the Expasy web server at https://swissmodel.expasy.org/ (accessed on 7 January 2026). This tool constructs protein homology models at various levels of complexity. Figures of the modeled AKAP4, RNF200, and SOX30 were generated with the Accelys Discovery Studio Visualizer software (V3).

In addition, the AlphaFold server (https://alphafoldserver.com/welcome, accessed on 7 January 2026), powered by AlphaFold 3, was employed to predict the three-dimensional structure of both wild-type and mutant proteins. It can generate highly accurate predictions of the structures of biomolecular complexes, including any chemical modifications of proteins.

To analyze the structural alterations induced by the identified mutation, we compared the non-covalent interactions between the wild-type and mutant protein models. Hydrogen bonds, ionic interactions (salt bridges), hydrophobic contacts, and van der Waals interactions were calculated and visualized using the AlphaFold server.

### 4.5. Hormone Measurements

Serum levels of reproductive and thyroid hormones (FSH, LH, testosterone, prolactin, and TSH) were measured using electrochemiluminescence immunoassay (ECLIA) in a certified clinical laboratory according to standard protocols.

## 5. Conclusions

In conclusion, we identified candidate genetic variants, including homozygous missense variants in *SOX30* and *RNF220*, as well as a hemizygous variant in *AKAP4*, that may contribute to NOA. The detected *SOX30* variant is predicted to have a possible impact on protein function and DNA-binding properties; however, this remains to be experimentally validated. The co-occurrence of *RNF220* and *AKAP4* suggests possible oligogenic contributions to male infertility. Identifying a variant in *AKAP4* in a patient with NOA also highlights the phenotypic variability of male infertility associated with this gene. Ultimately, our findings are consistent with a potential contribution of the identified rare variants to the genetic heterogeneity of NOA.

## Figures and Tables

**Table 1:** Hormonal evaluation of patients with non-obstructive azoospermia.

Parameter	Patient AZO-3	Patient AZO-8	Normal Range
FSH (mIU/mL)	13	7.4	1.5–13
LH (mIU/mL)	7.49	2.1	1.2–9
TSH (μIU/mL)	2.2	0.9	0.3–4.6
Testosterone (ng/mL)	1.94	2.4	2.8–8
Prolactin (ng/mL)	9.98	7.6	2.5–17

**Table 2:** Information of the identified potential disease-causing variants.

Patient ID	AZO-3	AZO-8	
Gene	*SOX30*	*RNF220*	*AKAP4*
Chromosomal Position (GRCh38)	chr5 157651180 G>A	chr1 44649687 C>T	chrX 50192359 T>C
mRNA/ cDNA Change	NM_178424.2 c.899C>T	NM_018150.4 c.1472C>T	NM_003886.3 c.2354A>G
Protein Change	p.(Ser300Phe)	p.(Thr491Met)	p.(Gln785Arg)
Zygosity status	Homo	Homo	Hemi
In silico Scores Mutation Taster Prediction	Deleterious	Deleterious	Deleterious
SIFT	Affect protein function (00.0)	Affect protein function (00.0)	Affect protein function (00.02)
PolyPhen-2	Probably damaging (1.00)	probably damaging (0.990)	Benign (0.322)
CADD	27.6	32	22.4
REVEL	Pathogenic (0.7)	Uncertain (0.57)	Benign (0.13)
AlphaMissense	Uncertain (0.34)	Uncertain (0.36)	Uncertain (0.48)
Alle frequency (gnomAD v4/ Number of homozygotes or hemizygotes)	0.00005908/0	0.00001505/0	0/0
dbSNP ID	rs375931805	rs746902232	–
KO–MGI (M)	MGI:1341157	–	–

## Data Availability

The data of this study are available upon reasonable request. The data are not publicly available due to privacy and ethical restrictions.

## Supplementary Materials

The following are available online at https://www.mdpi.com/article/10.3390/ijms27167230/s1.
